# The role of ferroptosis in lung cancer

**DOI:** 10.1186/s40364-021-00338-0

**Published:** 2021-11-06

**Authors:** Sikai Wu, Chengchu Zhu, Daolin Tang, Q. Ping Dou, Jianfei Shen, Xin Chen

**Affiliations:** 1 Department of Thoracic Surgery, Taizhou Hospital of Zhejiang Province affiliated to Wenzhou Medical University, Linhai, China; 2 Key Laboratory of Minimally Invasive Techniques & Rapid Rehabilitation of Digestive System Tumor of Zhejiang Province, Linhai, China; 3 Department of Surgery, UT Southwestern Medical Center, Dallas, TX USA; 4 Department of Oncology, School of Medicine, Barbara Ann Karmanos Cancer Institute, Wayne State University, Detroit, MI 48201 USA; 5 Departments of Pharmacology & Pathology, School of Medicine, Wayne State University, Detroit, MI 48201 USA; 6 Guangzhou Municipal and Guangdong Provincial Key Laboratory of Protein Modification and Degradation, School of Basic Medical Sciences, Affiliated Cancer Hospital & Institute of Guangzhou Medical University, Guangzhou, China

**Keywords:** Ferroptosis, Lung cancer, iron, Lipid peroxidation, ROS

## Abstract

Lung cancer is one of the most common cancers in the world. Although medical treatment has made impressive progress in recent years, it is still one of the leading causes of cancer-related deaths in men and women. Ferroptosis is a type of non-apoptotic cell death modality, usually characterized by iron-dependent lipid peroxidation, rather than caspase-induced protein cleavage. Excessive or lack of ferroptosis is associated with a variety of diseases, including cancer and ischaemia-reperfusion injury. Recent preclinical evidence suggests that targeting ferroptotic pathway is a potential strategy for the treatment of lung cancer. In this review, we summarize the core mechanism and regulatory network of ferroptosis in lung cancer cells, and highlight ferroptosis induction-related tumor therapies. The reviewed information may provide new insights for targeted lung cancer therapy.

## Introduction

Lung cancer is one of the most common causes of cancer-related death in the world. There are two major types of lung cancer: non-small-cell lung cancer (NSCLC) and small-cell lung cancer (SCLC), which account for approximately 85 and 15% of all newly diagnosed lung cancers, respectively [1]. Although the difference between NSCLC and SCLC is obvious, their pathological mechanisms are still unclear. While patients with early-stage lung cancer can undergo surgery, some type of local lung cancers can progress to invade the surrounding organs and tissues (e.g., heart, great vessels, trachea, and vertebrae). These tumors are classified as unresectable and are usually treated with palliative chemotherapy and/or radiation. Recently, immunotherapy has emerged as an alternative treatment option for patients with lung cancer. The programmed death 1 (PD-1) inhibitors nivolumab and pembrolizumab, along with the programmed death-ligand 1 (PD-L1) inhibitor atezolizumab, have been approved for the treatment of metastatic NSCLC which have improved the prognosis of some patients with NSCLC [2]. Despite advances in the development of new treatments, the overall 5-year survival rate of lung cancer patients is still about 16% [3]. Thus, novel therapeutic strategies for lung cancer are urgently needed.

Ferroptosis is a form of regulated cell death (RCD) that can be triggered by iron-dependent lipid peroxidation [4]. Ferroptotic cells have necrosis-like morphological changes, such as plasma membrane rupture and shrunken mitochondria with a reduced crista [5]. Generally, ferroptosis is highly dependent on the production of lipid reactive oxygen species (ROS), but without activation of caspases, mixed lineage kinase domain like pseudokinase (MLKL), and gasdermin D (GSDMD), which are key effectors or regulators of other forms of RCD (apoptosis, necroptosis, pyroptosis, respectively) [4]. Unlike the pro-survival effects of autophagy under many stresses, the core components of the autophagic machinery, including autophagy related (ATG) protein family, contribute to ferroptotic cell death [6]. Although ferroptotic response may play a dual role in regulating tumor initiation [7, 8], the induction of ferroptosis by small molecular drugs represents a potential anti-tumor strategy [9]. In this review, we aim to discuss the signal, mechanism, and function of ferroptosis in lung cancer, which may provide a potential strategy for the treatment of lung cancer.

### The mechanism and regulation of ferroptosis

**Fig. 1:**
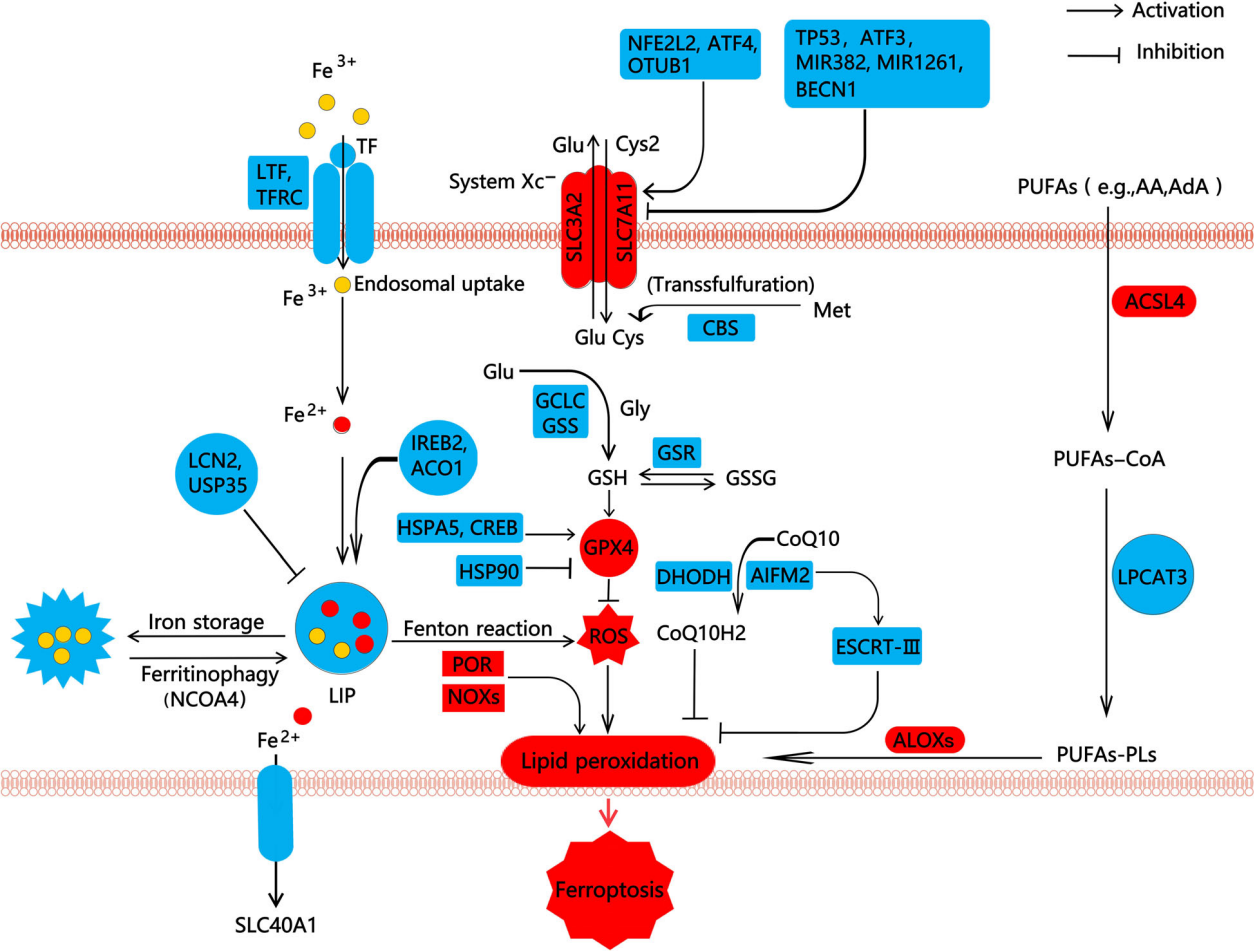
Core mechanism of ferroptosis. Ferroptosis is an iron-dependent form of regulated cell death. Fe^2+^ can participate in the Fenton reaction and induce lipid peroxidation. LTF and TF-mediated iron uptake and NCOA4-mediated ferritin degradation can promote ferroptosis, while the iron exporter SLC40A1 is a repressor of ferroptosis. GSH-dependent system (SLC7A11-GSH-GPX4 pathway) and GSH-independent system (CoQ, BH4, and ESCT-III pathway) inhibit ferroptotic cell death through suppressing lipid peroxidation or membrane damage. ACSL4, LPCAT3, and ALOX promote lipid peroxidation on the membrane. In addition, POR and NOXs also contribute to lipid peroxidation in a context-dependent manner. The complex regulation of SLC7A11 or GPX4 by multiple mechanism affects ferroptosis sensitivity (see text). Abbreviations: ACO1, aconitase 1; ACSL4, acyl-CoA synthetase long-chain family member 4; ALOXs, lipoxygenases; AIFM2, apoptosis-inducing factor mitochondria-associated 2; ATF3, activating transcription factor 3; ATF4, activating transcription factor 4; BECN1, beclin 1; CBS, cystathionine beta-synthase; CoQ10, coenzyme Q10; CoQ10H2, reduced coenzyme Q10; CREB, CAMP responsive element binding protein; Cys, cysteine; Cys2, cystine; DHODH, dihydroorotate dehydrogenase; ESCRT-III, endosomal sorting complexes required for transport-III; GCLC, glutamate-cysteine ligase catalytic subunit; Glu, glutamate; Gly, glycine; GPX4, glutathione peroxidase 4; GSH, glutathione; GSS, glutathione synthetase; GSR, glutathione reductase; GSSG, oxidized glutathione; HSPA5, heat shock protein family A member 5; HSP90, heat shock protein 90; IREB2, iron-responsive element binding protein 2; LCN2, lipocalin 2; LIP, labile iron pool; LPCAT3, lysophosphatidic transferase 3; LTF, lactotransferrin; LSH, lymphoid-specific helicase; Met, methionine; NFE2L2, nuclear factor erythroid 2-like 2; NCOA4, nuclear receptor coactivator 4; NOXs, NADPH oxidases; OTUB1, OTU deubiquitinase, ubiquitin aldehyde binding 1; POR, cytochrome P450 oxidoreductase; PUFAs, polyunsaturated fatty acid; PUFAs-CoA, polyunsaturated fatty acids-CoA; PUFA-PLs, phospholipids containing polyunsaturated fatty acids; ROS, reactive oxygen species; SLC3A2, solute carrier family 3 member 2; SLC40A1, solute carrier family 40 member 1; SLC7A11, solute carrier family 7 member 11; TF, transferrin; TFRC, transferrin receptor; TP53, tumor protein p53; USP35, ubiquitin specific peptidase 35

Ferroptosis is mainly driven by lipid peroxidation and is regulated at multiple levels, including glutathione (GSH)-dependent and -independent antioxidant pathways, the biological relevance of lipid peroxidation, and complex iron metabolism (Fig. 1).

#### GSH-dependent antioxidant pathway

The system xc^−^-GSH-glutathione peroxidase 4 (GPX4) pathway plays a central role in the regulation of ferroptotic cell death. GSH is important for intracellular redox balance. Consequently, the depletion of GSH will hinder GPX4-mediated antioxidant defense, leading to the accumulation of cellular lipid ROS, which is toxic to cell membranes.

System xc^−^ is an amino acid transporter with two key components, solute carrier family 3 member 2 (SLC3A2) and solute carrier family 7 member 11 (SLC7A11). System xc^−^ mediates the exchange of extracellular cystine and intracellular glutamate with a stoichiometry of 1:1. After entering the cell, cystine transforms into cysteine, which is an essential precursor for the production of GSH by glutamate-cysteine ligase catalytic subunit (GCLC) and glutathione synthetase (GSS). Preclinical studies have shown that small molecules (including erastin, sorafenib, glutamate, and sulfasalazine) induce ferroptosis by inhibiting the activity of system xc^−^ or SLC7A11 in vitro and in vivo [4, 10]. However, pharmacological inhibition of SLC7A11 by a small molecular compound, HG106 can induce a ROS-dependent, rather than ferroptosis-dependent, suppression of tumor growth in KRAS-mutant lung cancer cells [11], supporting a broad role of SLC7A11 in regulating ferroptotic and non-ferroptotic cell death [12]. Regardless, multiple evidence support SLC7A11 as a therapeutic target since it can promote lung cancer progression in vitro and in vivo [11, 13]. It is worth noting that the concept of ferroptosis may be derived and developed from “oxytosis”, which is a ROS-dependent cell death in nerve cells caused by glutamate-induced system xc^−^ inhibition [14].

Several prominent molecular mechanisms have been proposed to regulate ferroptosis by modulating the expression and/or activity of SLC7A11. At the transcriptional level, SLC7A11 is upregulated in cancer cells by transcription factors, such as nuclear factor erythroid 2-like 2 (NFE2L2/NRF2) [15] and activating transcription factor 4 (ATF4) [16]. SLC7A11 could be also downregulated by certain transcription factors, such as tumor protein p53 (TP53) [17] and activating transcription factor 3 (ATF3) [18]. At the post-transcriptional level, SLC7A11 is stabilized by OTU deubiquitinase via ubiquitin aldehyde binding 1 (OTUB1)-mediated deubiquitination in H1299 lung cancer cells [19]. At the epigenetic level, non-coding RNAs, such as MIR382 [20] and MIR1261 [21], can also reduce the expression of SLC7A11 to promote ferroptosis. Moreover, the phosphorylation of beclin1 (BECN1) mediated by AMP-activated protein kinase (AMPK) promotes ferroptosis by inhibiting the activity of system xc- and subsequent GSH production [22]. In addition to system xc-, the transsulfuration pathway provides additional resources for GSH maintenance by mediating cysteine synthesis. The cystathionine synthase (CBS) is a key enzyme of the activation of transsulfuration, acting as a ferroptosis suppressor [23].

GPX4, a selenium-containing enzyme, is found to directly quench membrane peroxidation by utilizing GSH. During this process, GSH is oxidized to GSSG, which is reconverted back to GSH by glutathione reductase (GSR). Ferroptosis can be induced by various GPX4 inhibitors, including RSL3 [24], FIN56 [25], and FINO2 [26], although their activities on ferroptosis may be different. Inhibition of upstream SLC7A11 suppresses GPX4 activity and also decreases its protein expression through either inhibiting its synthesis [27] or enhancing its protein degradation [28, 29]. The protein synthesis or degradation of GPX4 is tightly controlled by several mechanisms. For example, in addition to selenium, isopentenyl pyrophosphate (IPP) produced by the mevalonate pathway favors GPX4 protein synthesis [25, 30]. Heat shock protein family A member 5 (HSPA5, best known as BIP) is a molecular chaperone in the endoplasmic reticulum, which directly binds GPX4 to prevent its degradation, thereby inducing cancer cell resistance to ferroptosis [28]. However, heat shock protein 90 (HSP90) mediated-GPX4 degradation through chaperone-mediated autophagy (CMA) facilitates ferroptosis [29], highlighting that different HSP members play different roles in the regulation of GPX4 protein stability. Additionally, deubiquitinase inhibitor PdPT promotes proteasomal degradation of GPX4 [31], although the selective mechanism of autophagy or ubiquitin-proteasome system for GPX4 degradation is unclear. Moreover, cAMP responsive element binding protein (CREB) stimulates GPX4 transcription to inhibit ferroptosis in lung cancer cells [32]. Overall, these findings establish a complex network that controls the expression and activity of GPX4 in ferroptosis, in addition to the fact that GPX4 is also involved in the regulation of other non-ferroptotic cell death, such as apoptosis, necroptosis, and pyroptosis [33–35]. Further understanding of the structure, location, and function of GPX4 is essential for the development of cell death therapies for cancer patients.

#### GSH-independent antioxidant pathway

Several GSH-independent systems, such as apoptosis-inducing factor mitochondria-associated 2 (AIFM2)-coenzyme Q10 (CoQ10), dihydroorotate dehydrogenase (DHODH)-CoQ10, GTP cyclohydrolase 1 (GCH1)- tetrahydrobiopterin (BH4), and endosomal sorting complexes required for transport-III (ESCRT-III) membrane repair system, can defend against ferroptosis through distinct mechanisms. CoQ10 is a component of the mitochondrial electron transport chain and can act as an antioxidant in mitochondrial and plasma membranes. In addition to its pro-apoptotic role in mitochondria, AIFM2 also functions as an oxidoreductase on plasma membrane that reduces CoQ10 to its reduced form, ubiquinol, which traps free radicals to inhibit lipid peroxidation [36, 37]. This location-dependent transition from pro-apoptotic to anti-ferroptotic function is mediated by AIFM2 myristoylation [36, 37]. Another function of AIFM2 on plasma membrane responsible for inhibiting ferroptosis is its ability to repair cell membranes by activating the ESCRT-III mechanism [38]. Critically, AIFM2 mediates resistance to ferroptosis in lung cancer cells in vitro and in vivo [36]. Unlike AIFM2, which acts on the plasma membrane, DHODH (a flavin-dependent mitochondrial enzyme) suppresses ferroptosis by reducing CoQ10 to ubiquinol in mitochondria [39]. Although these findings indicate that the production of CoQ10 in different organelles has the ability to inhibit ferroptosis, a key unresolved question is whether this defense mechanism is tumor type-specific.

Moreover, BH4 functions as a cofactor for several enzymes (e.g., nitric oxide synthase [NOS]) and exerts potent antioxidant effects. GCH1 is the rate-limiting enzyme in the biosynthesis of BH4, thereby protecting cells against ferroptosis [40, 41]. In the final stage, the plasma membrane damage caused by the ferroptotic signal can be repaired by the ESCRT-III machine, which performs a topologically unique membrane bending and fracture reaction [42, 43]. Once the membrane repair ability is weaker than the continuous damage signal, ferroptotic death will be irreversible. The direct consequence of plasma membrane rupture is the release of cell contents, especially damage associated molecular patterns (DAMP) [44], which mediates the inflammatory response caused by ferroptotic cell death.

#### Lipid peroxidation

The accumulation of lipid peroxidation in cells represents the most typical metabolic characteristics of ferroptosis. The peroxidation of lipid in the biofilm can cause oxidative damage to the membrane and finally leads to cell death. Lipid peroxidation occurs mostly in polyunsaturated fatty acids (PUFAs), which contain multiple double bonds that are easily attacked by ROS. During lipid peroxidation, several enzymes involved in lipid metabolism act as positive regulators of ferroptosis. Acyl-CoA synthetase long chain family member 4 (ACSL4) promotes the synthesis of PUFA-CoA from PUFAs, such as arachidonoyl (AA) and adrenal (AdA), resulting in activation of PUFAs [45–47]. After ACSL4-driven esterification, lysophosphatidic transferase 3 (LPCAT3) fuses PUFAs into phospholipids, forming phospholipids containing polyunsaturated fatty acids (PUFA-PLs) [45]. Meanwhile, lipoxygenases (ALOX), non-heme iron-containing enzymes act as the main enzyme mediating in the direct oxidation of PUFAs during ferroptosis. This notion is supported by the findings that ALOX inhibitors (e.g., cinnamyl-3,4-dihydroxya-cyanocinnamate, baicalein, PD146176, zileuton, and AA-861) can effectively inhibit erastin- or RSL3-induced ferroptosis [48]. In addition, genetic inhibition of ALOX, including ALOXE3, ALOX5, ALOX12, and ALOX15, also inhibits ferroptosis in a context-dependent manner [48–52]. Notably, ALOX15 silencing suppresses ferroptosis induced by erastin and RSL3 in Calu-1 lung cancer cells [51], while ALOX12 and ALOX15 mediate TP53-dependent ferroptosis in H1299 lung cancer cells [52, 53]. These findings support that the ALOX family may play a cell-type-dependent role in different lung cancer cells. Although enzymes like cytochrome P450 oxidoreductase (POR) [54, 55] and NADPH oxidases (NOXs) [56, 57] also participate in lipid peroxidation during ferroptosis, their possible effects on lung cancer cells have not been determine.

#### Iron metabolism

Most of the intracellular iron exists in the form of Fe^2+^ in the free state or is stored in the form of Fe^3+^ in ferritin. Fe^2+^ reacts with hydrogen peroxide (H_2_O_2_) through Fenton reaction to generate hydroxyl radicals, which can attack PUFA on the membrane during ferroptosis. Ferritin is the main iron-storage protein and prevents Fe^2+^ from being oxidized by H_2_O_2_. In contrast, the degradation of ferritin by selective autophagy, called ferritinophagy, promotes ferroptosis in many cell models [58, 59], including lung cancer cells [60]. In fact, many cellular processes that can change any step of iron uptake, storage, utilization, and output may affect the sensitivity of cells to ferroptosis. For example, increased iron uptake by the transferrin receptor (TFRC) [61] or lactotransferrin (LTF) [62] promotes ferroptosis, whereas solute carrier family 40 members 1 (SLC40A1)-mediated iron export prevents ferroptotic cell death [63, 64]. Both autophagy and ubiquitin-proteasome system mediate SLC40A1 degradation in different conditions. In lung cancer cell, proteasomal degradation of SLC40A1 is inhibited by ubiquitin specific peptidase 35 (USP35), which is a deubiquitinating enzyme that is abnormally expressed in many cancers [65]. Moreover, lipocalin 2 (LCN2) inhibits ferroptosis by limiting iron uptake [66–68], while aconitase 1 (ACO1, also known as IRP1) and iron responsive element binding protein 2 (IREB2, also known as IRP2) promote ferroptosis in cancer cells by regulating the translation of iron metabolism-related protein [69]. However, the global view on the effect of iron metabolism in the body (e.g., iron absorption and tissue distribution) on ferroptotic response remains a secret.

### The regulation of ferroptosis in lung cancer

**Fig. 2:**
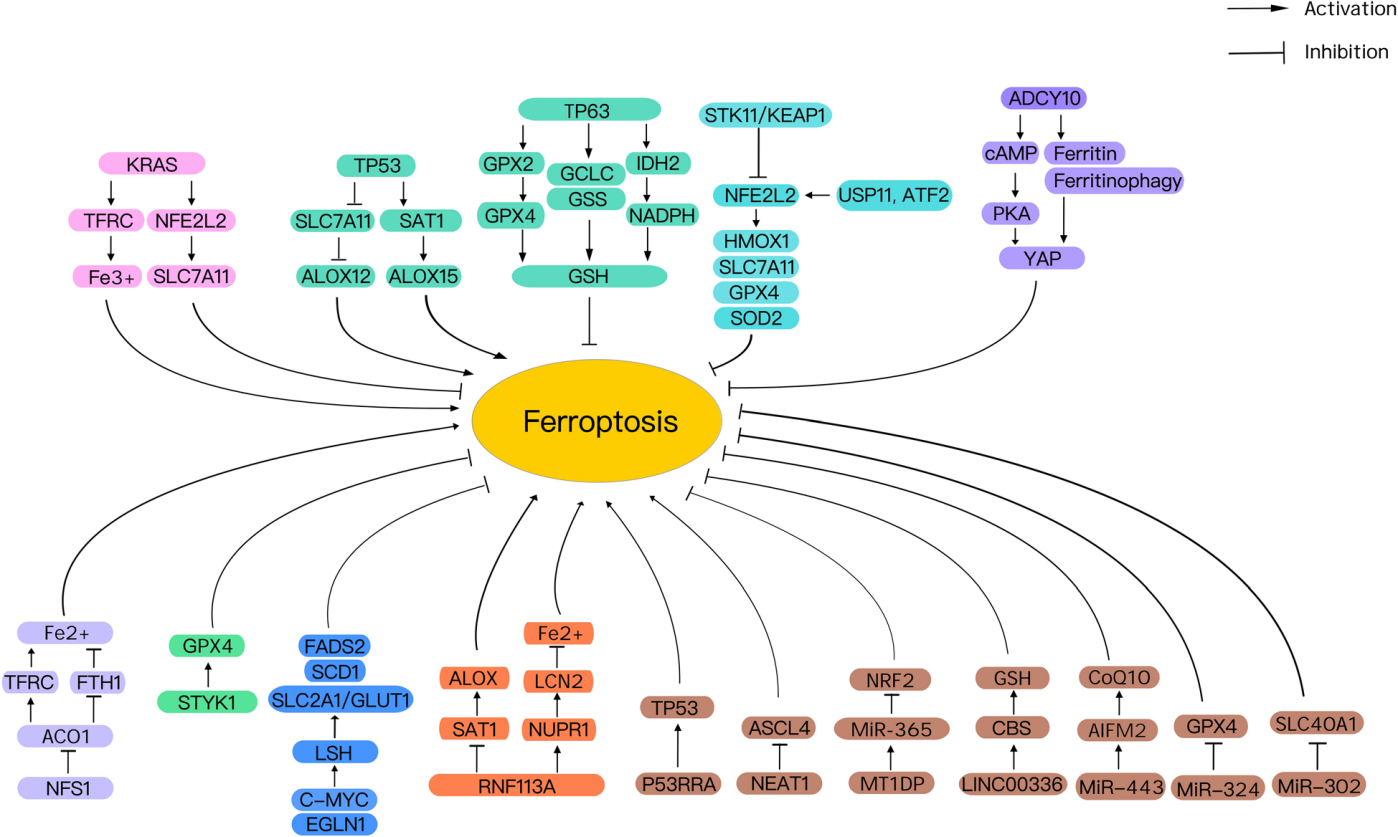
Key pathways in regulation of ferroptotic cell death in lung cancer. Several key regulators, such as KRAS, TP53, NFE2L2, YAP, NFS1, STYK1, LSH, RNF113A, and non-coding RNA play multiple roles in shaping ferroptosis sensitivity (see text). Abbreviations: ACO1, aconitase 1; ACSL4, acyl-CoA synthetase long-chain family member 4; ADCY10, adenylate cyclase 10; AIFM2, apoptosis-inducing factor mitochondria-associated 2; ALOX, lipoxygenases; ALOX12, lipoxygenases 12; ALOX15, lipoxygenases 15; cAMP, cyclic adenosine monophosphate; CBS, cystathionine beta-synthase; CoQ10, coenzyme Q10; FADS2, fatty acid desaturase 2; FTH1, ferritin heavy chain 1; GCLC, glutamate-cysteine ligase catalytic subunit; GSH, glutathione; GSS, glutathione synthetase; GPX2, glutathione peroxidase 2; GPX4, glutathione peroxidase 4; HMOX1, heme oxygenase 1; IDH2, isocitrate dehydrogenase 2; KEAP1, kelch like ECH associated protein 1; LCN2, lipocalin 2; LSH, lymphoid-specific helicase; MTIDP, metallothionein 1D pseudogene; NADPH, reduced form of nicotinamide-adenine dinucleotide phosphate; NEAT1, nuclear paraspeckle assembly transcript 1; NFE2L2, nuclear factor erythroid 2-like 2; NFS1, NFS1 cysteine desulfurase; NRF2, nuclear factor erythroid 2-related factor 2; NUPR1, nuclear protein 1; PKA, protein kinase A; SAT1, spermidine/spermine N1-acetyltransferase 1; SCD1, stearoyl-CoA desaturase 1; SLC2A1/GLUT1, solute carrier family 2 member 1; SLC7A11, solute carrier family 7 member 11; SOD2, superoxide dismutase 2; STYK1, serine threonine tyrosine kinase 1; TFRC, transferrin receptor; TP53, tumor protein p53; TP63, tumor protein p63; USP11, ubiquitin specific peptidase 11; YAP, yes associated transcriptional regulator

Cigarette smoking-induced chronic obstructive pulmonary disease (COPD) is one of the leading risks for lung cancer. Whole cigarette smoke condensates induce ferroptosis in human bronchial epithelial cells, contributing to the pathogenesis of COPD in lung cancer patients [70, 71]. These findings establish the first link between ferroptosis, COPD, and lung cancer. In addition, iron accumulation by ferritinophagy is involved in cigarette smoking-induced ferroptosis, accompanied by release of damage-associated molecular patterns (DAMPs) and pro-inflammatory cytokines from lung epithelial cells [71]. A bioinformatics prediction study reports that five ferroptosis-related genes (ALOX5, dipeptidyl peptidase 4 [DPP4], FA complementation group D2 [FANCD2], GCLC, and SLC7A11) may be involved in NSCLC progression and prognosis [72]. Further emerging evidence confirms regulation of ferroptosis by various pathways involved in lung cancer (Fig. 2). Altogether, these experimental results and bioinformatics analysis support the potential role of ferroptosis in lung tumorigenesis.

#### KRAS

KRAS mutations (especially KRAS-G12C) are commonly found in lung cancer and are associated with poor prognosis and therapy resistance. Historically, ferroptosis has been described as oncogenic RAS-dependent cell death, further supporting the idea that induction of ferroptosis is a target therapy. Consistent with this notion, two classical ferroptosis small molecule inducers, erastin and RSL3, are lethal chemical agents in KRAS mutation-containing tumor cells, including various lung cancer cells (e.g., Calu-1) [73, 74]. Moreover, KRAS mutant lung cancer cells are sensitive to ferroptosis induced by the inhibition of SLC7A11 [11]. The observations on that ferroptosis can be induced in both RAS-dependent and independent manners raise the question of the effect of genetic mutations on ferroptosis sensitivity. Several mechanisms have been proposed for the pro-ferroptotic death effect of KRAS. First, oncogenic RAS signaling increases cellular iron accumulation by increasing the expression of TFRC [74]. Secondly, KRAS mutation may increase NFE2L2-dependent SLC7A11 expression, providing more targets for SLC7A11 inhibitors [11, 75]. Since wild-type KRAS and mutant KRAS can affect each other in the context of mutant KRAS-driven tumors [76], it will be interesting to study the effect of this interaction on ferroptosis.

#### TP53

Tumor suppressor TP53 is a frequently mutated gene in various cancers, including lung cancer. In addition to its role in apoptosis, TP53 is an important regulator of non-apoptotic cell death, including ferroptosis. TP53 suppresses lung tumor growth by induction of ferroptosis [17]. Of note, the function of TP53 in ferroptosis is bidirectional, depending on its gene target or binding protein partner. For example, TP53 has anti-ferroptosis effects in colorectal cancer cells since it can inhibit DPP4-mediated NOX activation through protein-protein interaction [57]. The activation of the TP53 pathway in ferroptosis of lung cancer A549 lung cancer cells involves erastin-induced oxidative DNA damage [77]. Consequently, increased TP53 transcription causes inhibition of SLC7A11 expression, resulting in the depletion of GSH and then induction of ferroptosis in certain cancer cells, including H1299 [17]. The activity of TP53 in ferroptosis is dually regulated by its acetylation. In particular, TP53-3KR, a three acetylation site-defective mutant of TP53 that has no apoptosis-inducible effect, preserves the ability to induce ferroptosis by downregulating SLC7A11 in H1299 cells [17]. However, simultaneous absence of TP53-3KR and acetylation at K98 (namely TP53-4KR) causes a failure in ferroptosis induction in H1299 cells [78]. Notable, ALOX12 activation, but not GPX4 inhibition, acts as a direct downstream of TP53-mediated SLC7A11 inhibition for ferroptosis in H1299 cells [52]. TP53 also activates spermine N1-acetyltransferase 1 (SAT1), which promotes activity of ALOX15 to enhance ferroptosis in H1299 cells [53]. These findings highlight the molecular basis for TP53-mediated ferroptosis through different ALOX, which is regulated by different upstream signals.

The important role of TP53 in radiotherapy-mediated cancer cell ferroptosis has also been proposed. Radiotherapy activates TP53 function, thus inducing ferroptosis in many cancers, including lung cancer [79]. Ferroptosis inducers further enhance radiotherapy-mediated tumor suppression in TP53-mutant lung cancer patient-derived xenografts [79]. However, the contribution of radiotherapy-induced apoptosis under this condition is still undetermined.

TP63 and TP73 are two homologs of TP53. The ∆Np63α is an isoform of TP63 and is frequently amplified in lung cancer [80]. ∆Np63α restricts ferroptosis through transcriptional regulation of GSH metabolism-associated genes, including GCLC, GSS, glutathione peroxidase 2 (GPX2), and isocitrate dehydrogenase 2 (IDH2) [81]. As mentioned earlier, GCLC and GSS inhibit ferroptosis by increasing the production of GSH in the cell. GPX2 may function in regulating ROS by utilizing GSH to compensate for the lack of GPX4. IDH2, an enzyme in mediating the production of NADPH, is the rate-limiting factor for the regeneration of GSH. It will be interesting to explore the effects of TP53 and TP63 on GSH-independent anti-ferroptosis pathways.

#### NFE2L2

NFE2L2 is a key regulator of cytoprotective responses, mainly as a transcription factor to control expression of multiple defensive genes. Approximately one-third of patients with NSCLC harbor mutations of either NFE2L2 or its negative regulator kelch like ECH associated protein 1 (KEAP1) [82]. In response to ferroptosis activators (e.g., erastin and sorafenib), NFE2L2 dissociates from KEAP1, resulting in stabilization of NFE2L2 protein and translocation to the nucleus [83]. Activation of NFE2L2 negatively regulates ferroptosis in various cells (including lung cancer cells) by upregulating various target genes, e.g. heme oxygenase 1 (HMOX1) [84, 85], SLC7A11 [86], GPX4 [87], and superoxide dismutase 2 (SOD2) [87]. NFE2L2 activity is further enhanced by serine/threonine kinase 11 (STK11) and KEAP1 co-mutation, which induces expression of ferroptosis-protective genes, such as stearoyl-CoA desaturase (SCD) and aldo-keto reductase 1 C family 1/2/3 (AKR1C1/2/3) in lung cancer cells [88]. These data may be important for further evaluation of ferroptosis sensitivity in lung cancer cells that carry NEF2L2 and KEEP mutations in vivo.

The expression of NFE2L2 is also regulated by ubiquitin specific peptidase 11 (USP11) and activating transcription factor 2 (ATF2) in lung cancer cells. Mechanically, deubiquitinase USP11 inhibits ferroptotic cell death via stabilizing NFE2L2 protein in H1299 cells [89]. In contrast, USP11 depletion increases sensitivity to ferroptosis induction, which contributes to suppression of lung cancer cell proliferation [89]. ATF2 significantly inhibits ferroptosis-inducing effects of JQ1 through upregulation of NFE2L2 in A549 cells [90]. In this regard, pharmacological inhibition of NFE2L2 could be exploited in order to enhance ferroptosis in lung cancer treatment.

#### Yap

Yes associated transcriptional regulator (YAP) is a downstream transcription factor of Hippo pathway, which is essential for growth, invasion, and metastasis of many solid tumors, including lung cancer [91]. Accumulation of endogenous glutamate by erastin increases ferroptosis sensitivity by suppressing YAP expression in lung cancer cells [92]. Adenylate cyclase 10 (ADCY10) suppresses YAP and therefore sensitizes lung cancer cells to induction of ferroptosis; this is done through inhibiting ferritin by transcription or activation of ferritinophagy-dependent degradation and consequently elevating iron [92]. Mechanically, glutamate accumulation stimulates Ca^2+^-dependent activation of ADCY10, which mediates cAMP production and subsequent activation of protein kinase A (PKA) pathway, ultimately leading to the inhibition of YAP in lung cancer cells [92]. However, suppression of YAP may also promote ferroptosis resistance in certain cancer. Under high cell density conditions, increased cell-cell contact induces cancer cell resistance to ferroptosis by inactivation of YAP [93]. In contrast, overexpression of mutant YAP-S127A induces its nuclear retention and increases ferroptosis in HCT116 human colon cancer cells by inducing TFRC and ACSL4 expression [93]. In general, in order to develop a successful anti-tumor strategy via modulation of YAP-related ferroptosis, it is necessary to analyze both upstream and downstream signals of YAP.

#### NFS1

Iron-sulfur (Fe-S) clusters are ubiquitous, versatile cofactors and required for fundamental life processes. The initial sulfur mobilization step of biosynthesis of the Fe-S cluster is mediated by mitochondrial NFS1, which releases sulfur from cysteine. NFS1 is overexpressed in patients with lung adenocarcinoma, and upregulated NFS1 promotes growth of primary lung tumor cells in vitro [94]. In contrast, knockdown of NFS1 enhances anticancer activity of ferroptosis agents in lung cancer cells [94]. Mechanically, suppression of NFS1 induces ACO1-mediated iron-starvation response, resulting in enhanced translation of TFRC and decreased translation of ferritin heavy chain 1 (FTH1) [94]. Although these findings suggest that targeting iron utilization in mitochondria may alter the sensitivity to ferroptosis, the potential effect of NFS1 on iron-dependent ALOX deserves further study.

#### STYK1

Serine threonine tyrosine kinase 1 (STYK1, also known as NOK), a member of the receptor tyrosine kinases (RTKs) family, can promote metastasis of lung cancer through inducing epithelial-mesenchymal transition (EMT) by activating the AKT/glycogen synthase kinase 3 beta (GSK3B) pathway [95]. Elevated STYK1 expression is also correlated with poor prognosis of patients with non-small cell lung cancer [96, 97]. Preclinical discovery of STYK1 as a repressor of ferroptosis in lung cancer cells further supports that inhibition of STKY1 may be beneficial to lung cancer patients. Subsequent mechanistic studies have shown that overexpression of STYK1 up-regulates expression of GPX4 protein, resulting in decreased sensitivity of lung cancer SW900 cells to ferroptosis [97]. However, detailed mechanisms of how STYK mediates GPX4 upregulation remain unclear. One possibility is that STYK1-dependent degradation through ubiquitin-proteasome system, rather than autophagy [98], is involved in the regulation of GPX4 degradation.

#### LSH

Lymphoid-specific helicase (LSH) is a chromatin remodeling protein and a member of the SNF2 family. LSH is overexpressed in lung cancer tissues and is associated with poor prognosis in patients with lung cancer [99]. LSH interacts with WD repeat domain 76 (WDR76) and inhibits ferroptosis through activating metabolism-related genes, e.g., solute carrier family 2 member 1 (SLC2A1/GLUT1), SCD, and fatty acid desaturase 2 (FADS2) in vitro and in vivo [100]. SCD and FADS2 belong to the family of fatty acid desaturase that mediates generation of monounsaturated fatty acids (MUFAs) to competitively inhibit PUFAs-induced ferroptosis [101]. Consequently, inhibition of SCD and FADS2 promotes ferroptotic cell death in lung cancer [102]. In addition, SLC2A1-mediated glucose uptake may facilitate ferroptosis by increasing fatty acid synthesis. Although this hypothesis is confirmed in pancreatic cancer [102], it needs to be validated in lung cancer cells. Furthermore, expression of LSH could be mediated by inhibition of hypoxia inducible factor 1 subunit alpha (HIF1A) through egl-9 family hypoxia inducible factor 1 (EGLN1) coupling MYC signal [102]. These findings demonstrate that LSH-related pathways involve hypoxia and fatty acid metabolism for the regulation of sensitivity to ferroptosis in lung cancer cells.

#### RNF113A

Ring finger protein 113A (RNF113A) is an RNA-binding protein that participates in regulation of pre-mRNA splicing. RNF113A expression is upregulated in lung cancer, leading to resistance to DNA damage induced by cisplatin [103]. In contrast, loss of RNF113A can restore cisplatin sensitivity partly by inducing lipid ROS production and subsequent ferroptosis in A549 cells [103]. As a subunit of spliceosome, RNF113A promotes SAT1 splicing of multiple proteins, including SAT1 and nuclear protein 1 (NUPR1) [103]. However, non-lung cancer models have suggested that SAT1 or NUPR1 can act as a promoter or a repressor of ferroptosis by maintaining polyamine metabolism or increasing LCN2-mediated iron exporting, respectively [53, 66]. Whether RNF113A-mediated dual downstream effector models exist in lung cancer cells is still unknown. In addition, RNF113A can function as an E3 ligase to regulate protein ubiquitination [104], which may have a potential role in regulating ferroptosis.

#### Non-coding RNA

Several studies have shown that non-coding RNA, including long-chain non-coding RNA (lncRNA) and microRNA (miRNA) are regulators of ferroptosis in lung cancer. Prognosis of lung cancer patients is associated with several ferroptosis-related lncRNAs, such as C5orf64, LINC01800, and LINC00968 [105]. LINC00472/P53RRA functions as a tumor suppressor and is downregulated in multiple cancers, including lung cancer [106]. The cytosolic LINC00472 competes with TP53 for G3BP stress granule assembly factor 1 (G3BP1) interaction, leading to increased TP53 nuclear retention and ferroptosis [106]. In contrast, lncRNA nuclear paraspeckle assembly transcript 1 (NEAT1) or LINC00336 decreases sensitivity of ferroptosis by downregulating ACSL4 expression or increasing CBS expression in NSCLC cells [107, 108]. The expression of LINC00336 is positively regulated by ELAV like RNA-binding protein 1 (ELAVL1) during ferroptosis [108].

Similarly, miRNA plays a dual role in ferroptosis, depending on its targets. For example, MIR324 direct targets GPX4 and restores ferroptosis sensitivity in cisplatin-resistant A549/DDP cells [109]. In contrast, MIR4443 inhibits cisplatin-induced ferroptosis by regulating expression of AIFM2 in an m6A-dependent manner via methyltransferase like 3 (METLL3) [110]. Other miRNA target genes for ferroptosis in lung cancer cells include SLC40A1, which is regulated by MIRNA302 [111]. In addition, metallothionein 1D pseudogene (MT1DP) enhances ferroptosis in NSCLC cells via targeting the MIR365-NFE2L2 axis [112]. These findings further confirm the post-transcriptional regulatory mechanism of ferroptosis.

#### Potential correlation among different regulatory pathway

Different signaling pathways (e.g., KRAS, TP53, NFE2L2, YAP, and LSH pathways) often cross talk to each other, thereby establishing complex loops of regulatory networks to regulate ferroptosis. For instance, recent studies show that depletion of KRAS increases the levels of TP53 and promotes TP53 activation by a ROS-dependent pathway [113]. Mechanically, KRAS depletion down-regulates the expression of NFE2L2 and its transcriptional targets, leading to increased ROS levels [113]. Consistently, KRAS transcriptionally upregulates NFE2L2, which enhances GPX4 levels and contributes to gemcitabine resistance [114]. Mutant KRAS^G12D^ downregulates YAP in normal human mammary cells, and YAP inactivation is required for mutant KRAS-mediated initiation of tumorigenesis [115]. DNA methylation modifies activity of LSH and transcriptional activity of mutant TP53 proteins that can be regulated by the transcriptional regulator YAP [116, 117]. In addition, LSH suppresses TP53 ubiquitination and transactivates TP53 to regulate lipid metabolism [118], which may be in close association with ferroptosis. Therefore, a more detailed understanding of correlation among different regulatory pathways in lung cancer would be useful for ferroptosis-targeted therapies.

### Induction of ferroptosis as a potential novel treatment strategy for lung cancer

In addition to chemotherapy, accumulating evidence suggests that ferroptosis is involved in anticancer effects of radiotherapy and immunotherapy [10, 119–121]. Importantly, GPX4 is positively related to resistance of lung cancer cells to L-685458, lapatinib, palbociclib, and topotecan [122, 123], indicating that targeting ferroptosis may overcome therapy resistance. Consistently, ferroptosis inducers can inhibit growth of drug-resistant tumor, such as epidermal growth factor receptor (EGFR) inhibitor-resistant lung cancer cells [124]. Here, we discuss several agents that potentially induce ferroptosis in lung cancer cells (Table 1), apart from classical ferroptosis inducers (e.g., erastin and RSL3).

**Table 1:** Summary of ferroptosis-inducing agents in lung cancer

Agent	Target	Model	Mechanism	Refs
**Adagrasib**	KRAS	KRAS^G12C^-positive patients	Inhibition of KRAS^G12C^	9
**Ammonium ferric citrate**	GPX4	A549, HCC827, H1299, and NCI-H661 cells	Inhibition of GPX4-GSS/GSR-GGT axis	[125]
**BSO**	GSH	Neuroendocrine SCLC cells	Inhibition of the GCLC	129,130
**Celastrol**	ROS	HCC827, A540, and H1299 cells	Accumulation of damaged mitochondria and induction of PINK1/Parkin-dependent mitosis	[126]
**Cisplatin**	GSH	A549 cells	Depletion of GSH	125
**Cryptotanshinone**	ROS	A549 and NCI-H520 cells	Generation of the ROS and induction of lipid peroxidation	[127]
**Curcumin**	GSH	A549 and H1299 cells	Activation of autophagy	[128]
**Dihydroartemisinin**	ROS	NCI-H23 and XWLC-05 cells	Inhibition of the PRIM2-SLC7A11 axis	113
**Dihydroisotanshinone I**	GPX4	A549 and H460 cells	Downregulation of GPX4 expression	[129]
**Erastin**	SLC7A11	N5CP and A549 cells	Inhibition of NFE2L2-SLC7A11 pathway; upregulation and activation of TP53	77,86, [130]
**Erianin**	CaM	H460 and H1299 cells	Induction of Ca2+/CaM signaling	133
**Ginkgetin**	GSH	A549, NCIH460, and SPC-A-1 cells	Inhibition of SLC7A11 and GPX4; downregulation of GSH/GSSG ratio; disruption of the NFE2L2-HMOX1 axis	85
**6-Gingerol**	USP14	A549 cells	Inhibition of USP14 activity; upregulation of BECN1 and LC3-II/LC3-I	131
**Levobupivacaine**	ROS	A549 and A427 cells	Up-regulation of TP53	[131]
**Orlistat**	Lipid peroxidation	A549 and H1299 cells	Inhibition of the expression of FAF2	137
**Paracetamol**	ROS	A549 and H1299 cells	Inhibition of NFE2L2-HMOX1	84
**PdPT**	GPX4	A549 and H1299 cells	Induction of GPX4 degradation	31
**RSL3**	GPX4	H1650, HCC827, and PC9 cells	Inhibition of GPX4 activity	[132]
**Siramesine and Lapatinib**	ROS	A549 cells	Iron release from lysosomes and HMOX1 degradation by the proteasome system	[133]
**Sorafenib**	SLC7A11	N5CP and A549 sells	Inhibition of NFE2L2-HMOX1 pathway	86, [130]
**Sulforaphane**	GSH	NCI-H69, NCI-H82, and NCI-H69AR cells	Inhibition of SLC7A11	[134]
**Zinc**	ROS	A549 cells	Induction of lipid peroxidation	[135]

Abbreviations: BECN1, beclin 1; BSO, buthionine-sulfoximine; CaM, calmodulin; FAF2, fas associated factor family member 2; GCLC, glutamate-cysteine ligase; GGT, gamma-glutamyltransferase; GSH, glutathione; GSS, glutathione synthetase; GSR, glutathione reductase; GSSG, oxidized glutathione; GPX4, glutathione peroxidase 4; HMOX1, heme oxygenase 1; LC3-I, microtubule-associated protein light chain 3-I; LC3-II, microtubule-associated protein light chain 3-II; NFE2L2, nuclear factor erythroid 2-like 2; PdPT, palladium pyrithione; PINK1, PTEN induced kinase 1; PRIM2, DNA primase subunit 2; ROS, reactive oxygen species; SCLC, small cell lung cancer; SLC7A11, the solute carrier family 7 member 11; TP53, tumor protein p53; USP14, ubiquitin-specific peptidase 14

#### Cisplatin

Cisplatin has been used to treat multiple human cancers (e.g., lung cancer), and its ability to induce apoptosis has been widely studied. However, activity of cisplatin to induce ferroptosis has recently been reported. For example, cisplatin induces lipid ROS production and cell death in A549 cells, which is partially reversed by the ferroptosis inhibitor ferrostatin-1 [136]. Cisplatin-induced ferroptosis in A549 cells is related to inhibition of mitochondrial function, reduction of GSH-mediated GPX4 activity, and IREB2-mediated iron metabolism [136]. Among them, combination of cisplatin and GSH is considered to be the direct mechanism of cisplatin-induced ferroptosis [137], although the well-known function of cisplatin is to bind to DNA. Other studies have found that cisplatin-induced ferroptosis of lung cancer cells is further enhanced by the GPX4 inhibitor RSL3 or the bioflavonoid ginkgolide in an autophagy-dependent manner [85, 122]. These studies strengthen the notion that ferroptosis is an autophagy-dependent cell death.

#### PdPT

In addition to inhibitors of GPX4 activity (e.g., RSL3 [24] and ML120 [138]), agents that are able to induce GPX4 protein degradation may also trigger ferroptotic cell death. GPX4 protein can be degraded by autophagy or ubiquitin-proteasome system [139]. In the ubiquitin-proteasome pathway, GPX4 is first ubiquitinated by an E3 ligase and then degraded by the protease complex proteasome. The ubiquitination process of GPX4 can be reversed by a deubiquitinase, which is an enzyme that removes ubiquitin, thereby enhancing the stability of its protein substrate. Palladium pyrithione (PdPT) is a pan-deubiquitinase inhibitor and can induce ferroptosis and apoptosis in NSCLC cells through different mechanisms [31]. PdPT-mediated degradation of GPX4 is the cause of ferroptosis in A549 and H1299 cells [31]. However, the specific deubiquitinase involved in PdPT-mediated GPX4 degradation has not been identified. In other cases, HSPA5 can stabilize the GPX4 protein through its molecular chaperone function in ferroptosis [28], indicating an integrated mechanism that controls the degradation of GPX4.

#### BSO

Neuroendocrine SCLC can give rise to many non-neuroendocrine SCLC cells that are slow-growing and relatively chemoresistant. Non-neuroendocrine SCLC, but not neuroendocrine SCLC, is vulnerable to ferroptosis induction. This may be due to that ether lipid metabolism in non-neuroendocrine cells is higher than in neuroendocrine small cell lung cancer [140]. Buthionine sulfoximine (BSO) is an inhibitor of GCLC, the rate-limiting step in GSH biosynthesis. BSO induces lipid ROS and ferroptosis in mouse non-neuroendocrine, rather than neuroendocrine SCLC cells in vitro and in vivo [140]. Other studies have shown that BSO-enhanced ferroptosis could be detected in cancer cells or patient samples with lower GSH levels [141]. Thus, it would be interesting to determine whether there is any difference in GSH level between non-neuroendocrine and neuroendocrine SCLC cells.

#### 6-Gingerol

6-Gingerol is a phenolic compound naturally present in ginger and has potential antitumor effects. 6-Gingerol induces autophagy-dependent ferroptosis in lung cancer cell by inhibiting ubiquitin specific peptidase 14 (USP14) expression [142]. 6-Gingerol can increase intracellular iron levels and lipid peroxidation through a variety of mechanisms, such as enhancing nuclear receptor coactivator 4 (NCOA4)-mediated ferritinophagy, increasing expression of autophagy regulators (BECN1 and LC3II), or regulating ATF4 activity in A549 cells [142]. In addition to regulating autophagy, BECN1 has the ability to inhibit SLC7A11 [22]. Moreover, ATF4 act as a ferroptosis repressor by transcriptionally upregulating expression of SLC7A11 [16]. Therefore, it is expected that 6-Gingerol may cause GSH depletion.

#### Erianin

Erianin is a natural product from *Dendrobium chrysotoxum* [143]. By activating Ca^2+/^calmodulin-dependent pathway, anti-cancer activity of erianin is linked to ferroptosis induction in lung cancer cells [144]. In contrast, calmodulin inhibition by calmidazolium blocks erianin-induced ferroptosis in H460 and H1299 lung cancer cells [144]. Moreover, erianin can inhibit migration of lung cancer cells by inhibiting EMT [144]. Since influx of Ca^2+^ triggered by ferroptosis activates ESCRT-III-mediated membrane repair [42, 43], the hypothesis that blocking ESCRT-III will enhance erianin-induced ferroptosis remains to be verified.

#### Dihydroartemisinin

Dihydroartemisinin (DHA) is an artemisinin derivative used as an effective antimalarial drug. DHA can induce autophagy, apoptosis, and ferroptosis in various cancer cells in an iron-dependent manner [145–147]. Some preclinical studies show that DNA primase subunit 2 (PRIM2) is a negative regulator of DHA-induced ferroptosis in lung cancer cells (NCI-H23) through sustaining SLC7A11 expression [113], indicating that PRIM2 may plays a DNA primase-independent role in shaping ferroptosis. However, the underlying mechanism of DHA-induced PRIM2 downregulation in ferroptosis remains unclear [113].

#### Orlistat

Orlistat is a Food and Drug Administration (FDA)-approved bodyweight loss drug and has an activity of inhibiting fatty acid synthase (FASN). Orlistat induces ferroptosis in lung cancer cells (A549 and H1299) by repressing expression of Fas associated factor family member 2 (FAF2), a key factor regulating lipid droplet formation [148]. In liver cancer cells, lipid droplet formation is increased in early stage, but decreased in late stage of ferroptosis [149]. Functionally, enhancing tumor protein D52 (TPD52)-dependent lipid droplet formation prevents ferroptosis, whereas autophagic degradation of lipid droplets (known as lipophagy) promotes ferroptosis by increasing intracellular PUFA levels [149]. Thus, lipid droplet functions as an anti-ferroptotic organelle. Further studies are necessary to elucidate whether orlistat also promotes lipophagy in lung cancer cells.

#### Immunotherapy

In antitumor immune responses, CD8^+^ T cells are the main effectors to kill tumor cells. Two most representative immune checkpoint pathways, cytotoxic T-lymphocyte–associated antigen 4 (CTLA-4)/B7 and PD-1/PD-L1, play critical roles in T cell coinhibition and exhaustion, by which tumors are able to evade anti-tumor immunity. Immunotherapies such as PD-1/PD-L1 checkpoint blockades stimulate the immune system and have achieved significant progress in several kinds of tumours, including lung cancer. A recent report shows that blockade of PD-L1 plus CTLA-4 is able to inhibit tumor growth of B16 melanoma in a mice model through the induction of ferroptosis [10]. Mechanically, interferon γ (IFNγ) secreted by cytotoxic CD8^+^ T-cells induces ferroptotic cell death in cancer cells, through downregulation of system xc^−^ subunits (SLC7A11 and SLC3A2) [10]. As such, this is likely an important mechanism whereby immune checkpoint blockade eliminates tumors. Therefore, the enhanced antitumor capacity of ferroptosis inducer cyst(e) inase combined with checkpoint blockade by induce ferroptosis has been demonstrated in mouse ovarian tumor model [10]. Induction of ferroptosis might be associated with enhanced clinical response to immunotherapy. Human melanoma tissues with higher CD8^+^ T cell infiltration is characterized by lower levels of SLC7A11/SLC3A2, and higher ferroptosis response signature (a gene set that was upregulated by ferroptosis) [10]. Consistently, low expression of SLC3A2 is associated with good prognosis of patients with melanoma [10]. Compared to melanoma patients who did not benefit from PD-1 blockade, those who show clinical benefit have downregulated expression of SLC3A2 during treatment [10]. Moreover, ferroptosis is found to be immunogenic in vitro and in vivo [150, 151]. Damage-associated molecular patterns (e.g., ATP and HMGB1) can be released from ferroptotic cells and act as immunogenic signal to activate the immune system [150]. Another study suggests that oxygenated phosphatidylethanolamines (PEs) mediate the phagocytosis of ferroptotic cells by binding to toll like receptor 2 (TLR2) on macrophages [152]. These advances indicate that ferroptosis has great potential to enhance the immunotherapy in cancer treatment, but the anti-tumour effect of immunotherapy-mediated ferroptosis remains to be confirmed in lung cancer model.

## Conclusions and perspectives

Like other types of cell death, ferroptosis plays important roles in both lung cancer development and therapy. Although great progress has been achieved in understanding the process and function of ferroptosis, there are still some challenges in translational medicine. First, several studies have attempted to discover molecular biomarkers to predict the ferroptosis response. These includes biochemical hallmarks (e.g., lipid peroxidation), genetic hallmarks (e.g., upregulation of NFE2L2), and protein hallmarks (e.g., protein degradation of GPX4) [153]. Since lipid peroxidation also occurs in other forms of RCD, it is still difficult to distinguish ferroptosis from non-ferroptotic death in vivo. Similarly, ROS-related cell death may be associated with alterations of genes and proteins similar to ferroptosis. Thus, there is no single specific marker to identify ferroptosis in vivo. In the future, identification of sensitive and specific biomarkers or assay may facilitate the application of ferroptosis-related therapy in cancer patients. Combing multiple modalities of biomarkers may also help in applying these biomarkers to guide ferroptosis-based treatment. Secondly, although many preclinical agents can specifically induce ferroptosis, none of them have entered clinical trials. Among which, SLC7C11 and GPX4 inhibitors are the most well established ferroptosis inducers. Because the mouse knockout model of slc7a11 is healthy in appearance and fertile [154] and global GPX4 knockouts mice display embryonically lethal in mice [155], it is speculated that SLC7A11 inhibitors may show higher safety than GPX4 inhibitors. However, as mentioned previously, SLC7A11 inhibitor HG106 can induce non-ferroptotic cell death in lung cancer cells, while GPX4 inhibitors appear to induce cancer cell-specific ferroptosis. Nevertheless, further study of the specificity and effects of targeting SLC7A11 or GPX4 in lung cancer in conditional knockout mice is needed. In addition, identifying novel regulators of ferroptosis is benefit for developing strategies for inducing ferroptosis in lung cancer. Given the important role of KRAS and TP53 in regulate ferroptosis, it remains to be determined the anti-cancer effects of ferroptosis-inducing agents in combination with clinical KRAS- or TP53-targeting drugs in lung cancer. For instance, KRAS inhibitor sotorasib/AMG510 has been approved by the FDA to treat patients with NSCLC, while TP53-activating drug APR-246 is currently tested in phase trials in patients with cancer. Some drugs, such as sulfasalazine, sorafenib, zalcitabine, and cisplatin, can be repurposed to induce ferroptosis. However, these drugs may also induce other types of RCD, which raises the urgency for the development of ferroptotic agents with high specificity and low cytotoxicity. Thirdly, due to the heterogeneity of tumors (including lung cancer), how to develop personalized treatment will be an active research area in the next few years. Overall, we need multidisciplinary cooperation to further explore the pros and cons of targeted ferroptosis, find new drugs, and evaluate potential clinical applications.

## Data Availability

Not applicable.
